# Synthesis of silver nanoparticles from *Vicia faba* aqueous extract with cytotoxic activity against human acute T cell leukemia

**DOI:** 10.1038/s41598-025-03679-0

**Published:** 2025-09-30

**Authors:** Elizabeth Martínez-Becerril, María G. González‑Pedroza, Antonio Sandoval-Cabrera, Raúl A. Morales‑Luckie, Pedro Estanislao Acuña-Ávila

**Affiliations:** 1 Facultad de Ciencias, Universidad Autónoma del Estado de México (UAEMex), Toluca, México; 2 Facultad de Medicina, Universidad Autónoma del Estado de México (UAEMex), Toluca, México; 3 Hospital para el Niño, Instituto Materno Infantil del Estado de México (IMIEM), Toluca, México; 4 Centro Conjunto de Investigación en Química Sustentable UAEMex-UNAM (CCIQS), Toluca, México; 5 Universidad Tecnológica de Zinacantepec (UTZIN), Zinacantepec, México

**Keywords:** Green synthesis, Silver nanoparticles, Cytotoxicity, Leukemia, Apoptosis, Cancer therapy, Biotechnology, Materials science, Nanoscience and technology

## Abstract

Silver nanoparticles (AgNPs) are among the most extensively utilized nanomaterials in commercial and biomedical applications due to their potent cytotoxic properties. Leukemia remains one of the most prevalent cancers worldwide, driving the search for novel therapeutic approaches. In this study, aqueous extracts from Vicia faba *seed coats* were employed for the green synthesis of AgNPs, and their anticancer activity was evaluated in vitro. The biosynthesized AgNPs exhibited an average hydrodynamic diameter of 23.14 ± 0.20 nm, with all particles measuring under 40 nm, and showed a characteristic surface plasmon resonance (SPR) peak at 430 nm. X-ray diffraction (XRD) analysis confirmed the crystalline nature of the nanoparticles, revealing distinct peaks corresponding to the (111), (200), (220), and (311) planes of metallic silver. Fourier-transform infrared spectroscopy (FTIR) indicated the presence of phenolic functional groups from the extract, likely involved in nanoparticle formation and stabilization. Cell viability assays demonstrated a dose-dependent cytotoxic effect on leukemia cells, with an IC₅₀ of 2.27 mg/mL. Apoptosis induction was confirmed by flow cytometry using the Annexin V assay, which revealed a significant increase in apoptotic cell populations with increasing AgNPs concentrations. Additionally, an increased percentage of cells in the sub-G1 phase was observed, further supporting apoptotic activity. At a concentration of 3 mg/mL, the AgNPs significantly reduced cell viability. These findings suggest that the proposed one-step, cost-effective biosynthesis method yields AgNPs with promising anticancer properties, highlighting their potential as a therapeutic tool for leukemia treatment.

## Introduction

Cancer represents a global challenge, impacting public health and economic worldwide^1^. According to the American Cancer Society (ACS) around 20 million new cancer cases were diagnosed in 2022 with 9.7 million deaths worldwide^2^. Leukemia ranked as the 13th most diagnosed cancer and the 10th leading cause of cancer-related deaths globally^2^. Additionally, 487,000 new cases of leukemia were diagnosed in 2022, ranking as the predominant^3^ cause of mortality among hematologic malignancies with an estimated of 305,000 deaths in the same year^2,3^. Chemotherapy^4,5^, radiation^6^, immunotherapy^7^, and hormone therapy^8^ represent conventional modalities employed in cancer treatment. However, despite their efficacy, these approaches are associated with significant adverse effects in patients. Furthermore, the current therapeutic landscape for leukemia lacks definitive preventive measures and treatments. Compounding this challenge is the propensity of many therapeutic agents to induce drug resistance^9^, thereby limiting their long-term effectiveness. Therefore, the exploration of novel therapeutic approaches for cancer therapy, pursuing improved delivery systems and efficient controlled release of treatments is a key aspect in treating leukemia.

Recently, nanotechnology has emerged as a promising platform for the treatment of leukemia^9,10^. By manipulating materials at the nanoscale, novel properties are unlocked, offering potential applications across various fields, particularly in medicine. Nanoparticles (NPs) exhibit significant influence over their optical, catalytic, and electronic properties, which can be tailored based on factors such as size, shape, and crystalline structure^11^. This versatility makes nanoparticles a compelling strategy for leukemia treatment, as they can be engineered to specifically target cancer cells, deliver therapeutic agents more efficiently, and minimize off-target effects. Notably, nanoparticles offer certain advantages, including the ability of penetrating tissues through fine capillaries and crossing the epithelial lining of the cancer cells^12^. Among these, silver nanoparticles (AgNPs) have emerged as a novel approach for cancer treatment, including leukemia^12,13^. Nanoparticles selectivity in targeting the cancer cells, helps minimize drug toxicity towards normal cells^12^, enhancing the overall safety and efficacy of treatment regimens.

Nanoparticles generation is achieved by involving different physical and chemical techniques. Where, both methods frequently employ radiation and toxic reductants and capping agents capable to affect humans and other living organisms^11^. Chemical synthesis of NPs is a method that enables control over nanoparticle size and can be conducted utilizing various chemical precursors within the reaction. A significant advancement in NPs synthesis lies in the adoption of green synthesis methods. This technique utilizes plant extracts, fungi, bacteria, and other biological resources as reducing agents, offering an economically viable and environmentally^13^ friendly method for NPs generation. Plant extracts are rich in phytochemicals such as terpenes, alkaloids or polyphenols that facilitate the reduction of metal ions and at the same time these bioactive compounds have potential anticancer applications^13–15^.

Faba bean (*Vicia faba* L.), a nutritious leguminous cool tolerant crop, is widely cultivated throughout the world^16^. According to FAOSTAT (2018), faba bean is the fourth most widely grown cool season legume after pea (*Pisum sativum*), chickpea (*Cicer arietinum*) and lentil (*Lens culinaris* Medik.)^17–19^. Faba bean seeds are an essential source of macronutrients such as proteins, mineral elements, and secondary metabolites including polyphenols^20^. Legumes boast a wealth of phytochemicals that have been demonstrated that poses anticancer activity^20^. Some of these compounds include antioxidants and phenols such as cyanogen and tannins, which are mainly located in the seed coat^16,21^. Seed coats are discarded when dry faba bean seeds are consumed, however it comprises a rich source of bioactive compounds that have been reported in inducing apoptosis in cancer cells, including acute leukemia^22,23^. Thus, we decided to use *V. faba* seed coat extract to enhance the health benefits of this plant. The synthesis of AgNPs from extracts obtained from different plants, and even from different parts of the plant, is very common and has been extensively studied^24–27^. However, the synthesis of silver nanoparticles from the seed coat of *V. faba*, which are a waste material, and their report is very infrequent, since the seed coats are very little exploited and have been found to possess better properties than the other parts of the *V. faba* plant. In addition, there are no reports on the morphology, size, crystallinity of the nanoparticles that can be generated from *V. faba* seed coat. Therefore, the present investigation aimed to (i) biosynthesis of silver nanoparticles (AgNPs) using *V. faba* seed coat extract as a bio-reductant and stabilizing agent; (ii) characterization of synthesized AgNPs using UV–visible spectroscopy, TEM, FTIR, DRX and DLS; (iii) determination of cytotoxic response of AgNPs against human leukemia cells (Jurkat cell line) and (iv) the mechanisms involved in AgNPs-induced Jurkat cell death by measuring cell cycle arrest, and apoptosis.

## Methods

### Synthesis and characterization of silver nanoparticles

Dry seeds of *V. faba* beans (*Diamante* variety) were obtained from the Instituto de Investigación y Capacitación Agropecuaria, Acuícola y Forestal of Mexico State, the use, management and collection of the species was carried out in accordance with Mexican guidelines and regulations. The seed coat was separated from the cotyledons and milled to obtain a size particle around 0.5 mm. To generate the extract, testa powder was boiled at 100 °C using distilled water (1:100) for 5 min. Then it was filtrated using Whatman No. 1 filter and stored for particle synthesis.

Silver nanoparticles were synthesized by applying a *bottom-up* method, using *V. faba* water extract as a reducing agent and as an oxidizing agent a solution of silver nitrate (AgNO_3_). In a volumetric flask, 50 mL of 1 mM AgNO_3_ solution were mixed with 50 mL of water extract to observe the reduction of Ag^+^ ions into Ag^0^. The reaction required exposure to light for at least 10 min to observe a color change confirming the complete reduction of Ag^+^ ions. The extract and nanoparticles solution were stored at 4 °C for further use.

#### UV–Vis spectroscopy

Reduction of silver ions from the colloidal mixture of AgNPs was analyzed by the UV-Visible absorption spectra as a function of time. UV-Vis spectrum was measured every 10 min until 6 h in the range 300–700 nm using the VELAB brand Ultraviolet visible spectrophotometer (VE-5100UV, USA).

#### Transmission Electron microscopy (TEM)

The TEM analysis was performed to characterize morphologically the nanoparticles using a JEOL-2100 Transmission Electron Microscope operating at 200 kV used with filament of LaB_6_. A drop of the AgNPs sample was placed on a rack until dry, and it was subsequently introduced to the sample holder and assembled in the equipment. TEM images of AgNPs were processed with the ImageJ software to obtain the diameter size distribution of particles.

#### FTIR spectroscopy

The infrared fingerprints of *Vicia faba* extract and AgNPs were obtained by using the Fourier Transform Infrared Spectroscopy (FTIR) spectrometer (IRPrestige-21, Shimadzu) in transmittance mode and using spectral range of 4000 –380 cm^− 1^. FTIR analysis was used to determine the plant compounds involved in the reduction of silver ions. Nanoparticles and extract were dried at room temperature and the dried powder was plated and analyzed.

#### Dynamic light scattering (DLS)

The hydrodynamic mean diameter was determined by photon correlation spectroscopy (PCS), using a 4700 C light-scattering device (Malvern Instruments, London, UK) with a He-Ne laser (10 mW). The light scattered by the samples was detected at 173°, and the temperature was set at 25 °C. The diffusion coefficient measured by dynamic light scattering was used to calculate the size of the silver nanoparticles obtained from the aqueous extract of *V. faba* by the Stokes-Einstein equation. The homogeneity of the size distribution was expressed as polydispersity index (PDI), which was calculated from the analysis of the intensity autocorrelation function.

#### X-Ray diffraction (XRD)

The x-ray diffraction pattern was performed in a range from 5 to 80°, operating at 30 kV and 25 mA, with a step size of 0.029193° and step time of 56.7 s in a Bruker D8 Advance powder diffractometer with Bragg-Bretano geometry, CuKa radiation and Linxeye detector.

### In vitro anticancer activity

#### Cell viability test

The impact of AgNPs and crude extract of *V. faba* was evaluated in vitro, using the stablished Acute T cell line-Jurkat, obtained from the American Type Culture Collection (ATCC, Rockville, MD, USA). The cell line was maintained in RPMI-1640 medium, supplemented with 10% fetal bovine serum (FBS), 100 U/mL of penicillin and 100 µg/mL of streptomycin (Sigma-Aldrich, St Louis, MO, USA) according to the ATCC protocol. Cells were cultured and grown at 37 °C in an incubator with a humidified atmosphere at 5% CO_2_ atmosphere with increased humidity.

Jurkat cells were cultured in 48-well plate and five different concentrations of AgNPs, and water extract were added: 0.375, 0.750, 1.5. 3 and 6 mg/mL. Samples without AgNPs or extract served as controls. After incubation for 24 h viability was determined using trypan blue dye-exclusion test. The assay was performed in triplicate. Results were calculated as viable cells in AgNPs, or extract treated cultures relative to control. The half minimal inhibitory concentration (IC50) was calculated by interpolation from dose–response curves using GraphPad Prism Software Version 8.0.

#### Annexin V-FITC assay

The evaluation of apoptotic or necrotic death was carried out using a commercially available Annexin V-FITC apoptosis detection kit (Sigma-Aldrich, ST, Missouri, USA), in accordance with the manufacturer’s protocol. Jurkat cells were cultured for 24 h in the presence of 0.75, 1.5 and 3 mg/mL for the AgNPs and 6 mg/mL of *V. faba* extract, untreated cells were used as controls. The test was performed as follows: after nanoparticles treatment, 1 × 10^5^ cells were suspended in 100 µL of Annexin binding buffer (1×). Next, 1 µL of Annexin V-fluorescein isothiocyanate (FITC) conjugate and 2 µL of Propidium Iodide (PI) were added to the samples. After 15 min of incubation in the dark, 400 µL of binding buffer (1×) were added to each test tube. Data were acquired with Gallios cytometer (Beckman Coulter). Cells were defined as early apoptotic, late apoptotic, and necrotic based on the proportion between FITC (Ex = 488 nm; Em = 350 nm) and/or PI (Ex = 488 nm; Em = 560–680 nm) fluorescence. Cell death analysis was measured with Gallios Analysis Software.

#### Cell cycle analysis

The Jurkat cells (2 × 10^5^ cells/mL) after 24 h of AgNPs treatment (0.75, 1.5 and 3 mg/mL) were suspended in ice-cold 70% ethanol for permeabilization during 30 min at room temperature. Before the analysis, the cells were washed using 1X PBS and incubated with 100 µL propidium iodide (PI) solution (50 µg/mL propidium iodide, RNAse (4 KU/mL), < 0.1% NaN_3_, saline and stabilizer) under dark conditions for 30 min. The cell cycle distribution was determined by WATSON MODEL in Kaluza analysis software version 1.3.

## Results and discussion

### UV–Vis analysis of AgNPs

**Fig. 1:**
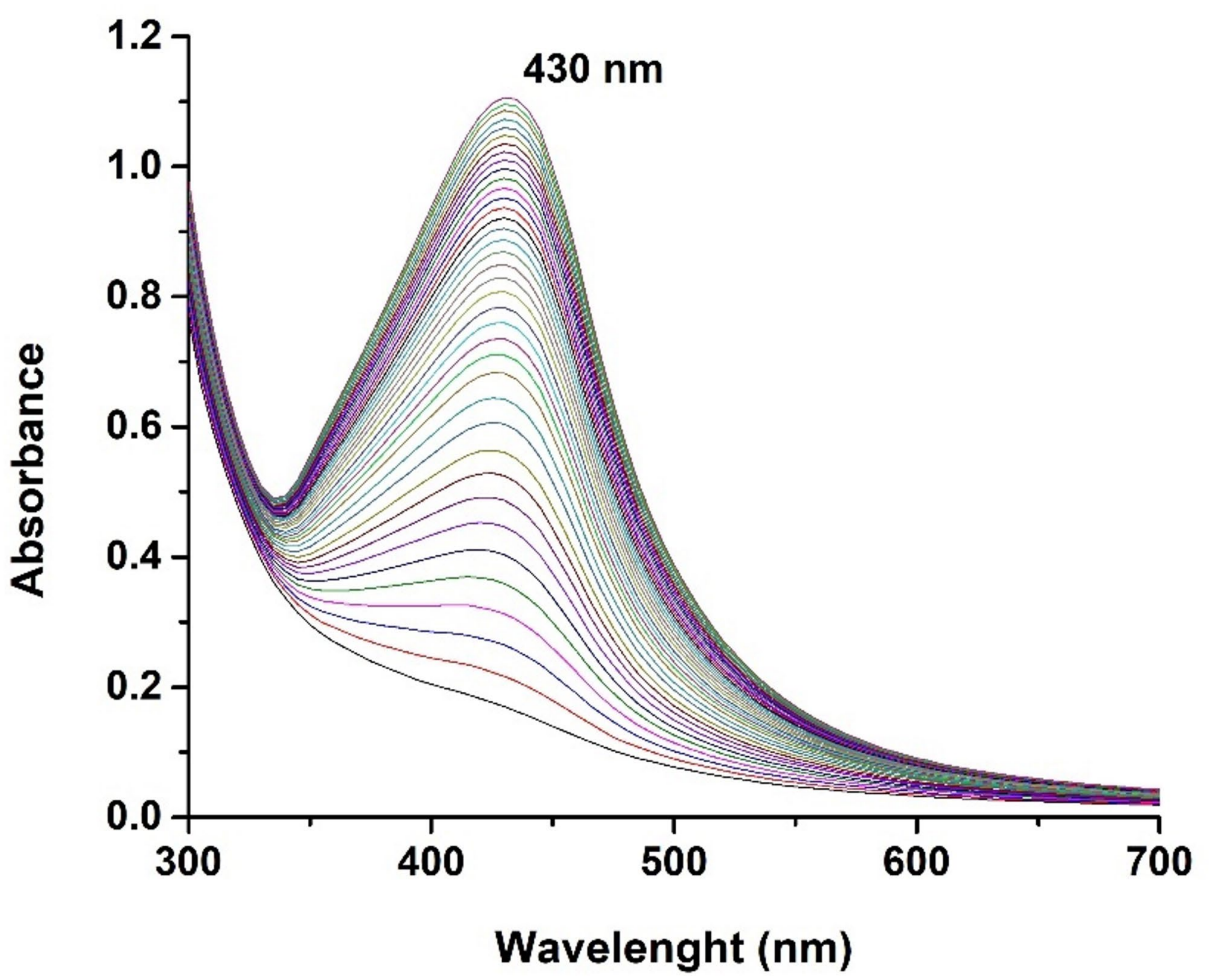
UV–Vis spectra of AgNPs biosynthesized with *Vicia faba* seed coat extract with a SPR peak at 430 nm.

An apparent color change from light yellow to yellowish-brown was noticed over the reaction period, ultimately reaching colloidal brown. This change of color is associated with nanoparticle synthesis reaction^28^. As the color intensity no longer changed, it is inferred that the reaction was terminated, indicating the generation of AgNPs, since this transformation is due to a redox reaction of the Ag^+^ metal silver ions on Ag° silver nanoparticles through the active molecules present in the extract^29^.The UV-Vis spectrum of the silver nanoparticles was tracked over a six-hour period at 10-minute intervals as showed in Fig. 1. The analysis revealed a prominent Surface Plasmon Resonance (SPR) peak at 430 nm, just within the spectral range of nanometric silver.

According to previous reports, a UV-Vis absorption band around 400–450 nm is an indication of SPR of AgNPs^28,30^. Additionally, our examinations of plasmon resonances over time suggest the polydisperse nature of the nanoparticles obtained^30^. Normally the green synthesis offers some polydispersity of nanoparticle size due to the multiple compounds possessing reducing agents, however, the results show a narrow band which according to the literature refers to a smaller size distribution^31,32^. This feature improves its suitability for medical applications. Furthermore, the observed maximum absorption band aligns with the characteristics of spherical nanoparticles^30^.

### Particle morphology and size of AgNPs

**Fig. 2:**
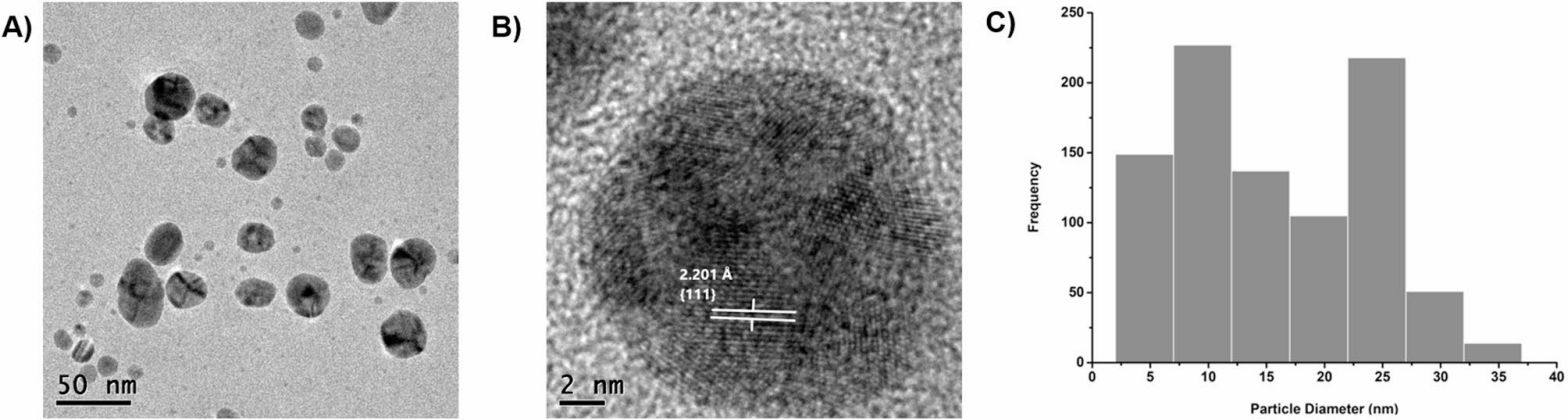
Size distribution and morphology determination of *V. faba* seed coat extract mediated silver nanoparticles. (**A**) TEM micrography of AgNPs. (**B**) HRTEM micrography. (**C**) Size distribution histogram of AgNPs.

The morphology, including the size and shape, of the green synthesized AgNPs using *V. faba* extract was determined through electron microscopy. The analysis of TEM images revealed that the AgNPs exhibited almost spherically shaped and suggest polydisperse behavior (Fig. 2A). Green synthesis usually shows quasi-spherical nanoparticles due to the compounds present in their structure. It should be noted that this synthesis method does not have a large environmental impact, since toxic compounds such as reducing agents, pH regulators, elevated temperatures and sophisticated equipment are not required compared to other methodologies^33,34^. Figure 2B depicts the High-Resolution Transmission Electron Microscopy (HRTEM) image of biosynthesized silver nanoparticles showing the lattice fringes quite clearly, with an interplanar distance of about 2.201 Å that could be associated with the (111) planes of silver, taking as reference the JCPDS card 00-004-0783.

The diameter measurement showed that the obtained nanoparticles ranged in size between 5 and 40 nm, the approximate size of nanoparticles was calculated to be 16 nm (Fig. 2C). The colloidal solution of nanoparticles was subjected to Dynamic Light Scattering (DLS) to determine their hydrodynamic size, and it was found to be 23.14 nm ± 0.20 nm.

### X-ray diffraction (XRD)

**Fig. 3:**
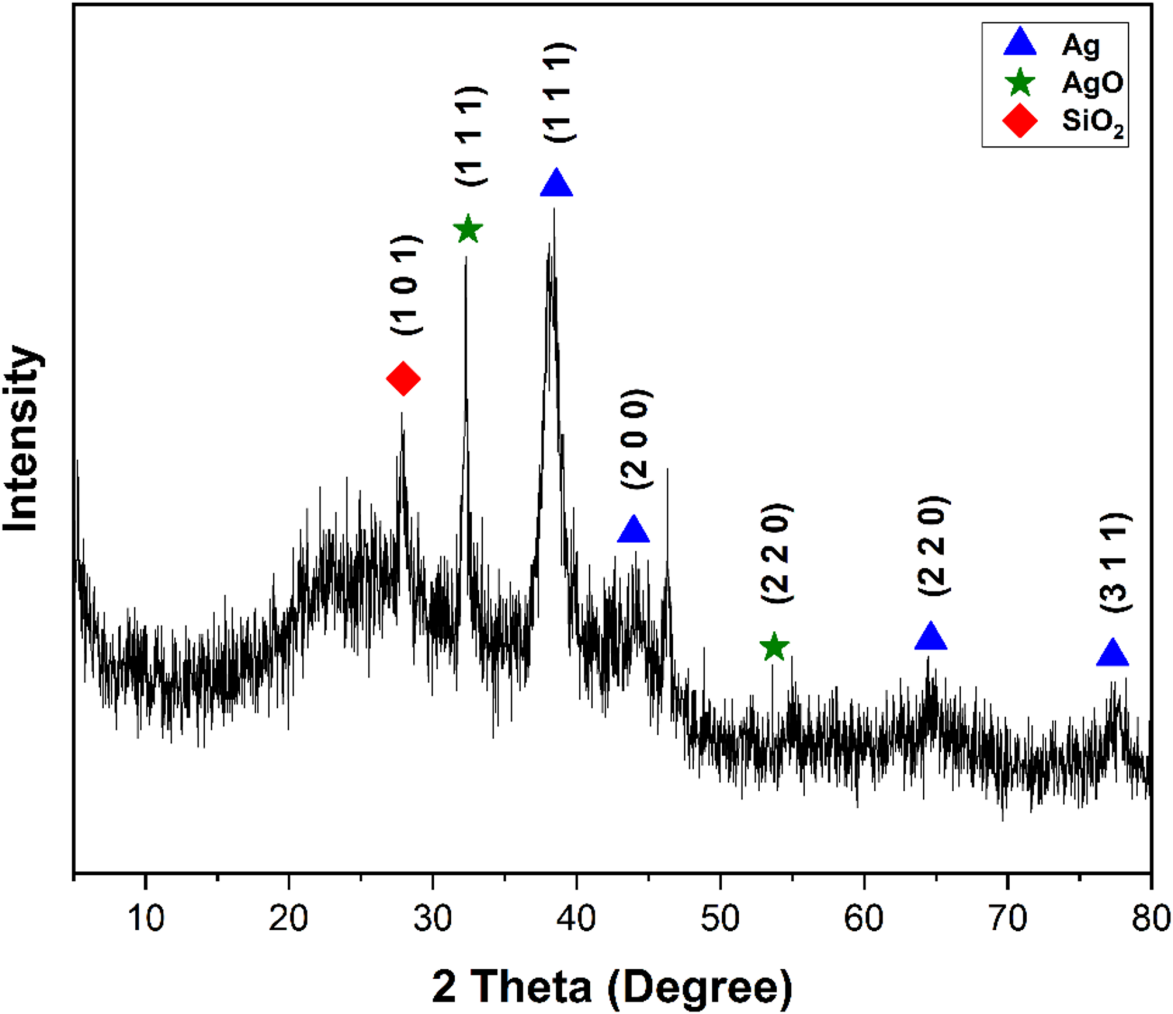
Diffractogram of AgNPs from *Vicia faba* seed coat.

X-ray diffraction spectroscopy (XRD) was used to identify the crystallinity of AgNPs. In the diffractogram, the diffraction planes (111), (200), (220) and (311) corresponding to metallic Ag were assigned to the AgNPs (Fig. 3).

However, the appearance of the other peaks is attributed to the crystalline diffraction planes (111) and (220) of the silver oxide, which could be due to the surface coating of the AgNPs or organic compounds present in the *V. faba* extract that act as reducing agents and are formed at the time of sample preparation. This is consistent with the FTIR and UV-vis spectra. On the other hand, a peak coinciding with the crystalline plane (101) appears, which corresponds to SiO₂. This is due solely to the slide that was used as support for the sample.

### FTIR analysis of AgNPs

**Fig. 4:**
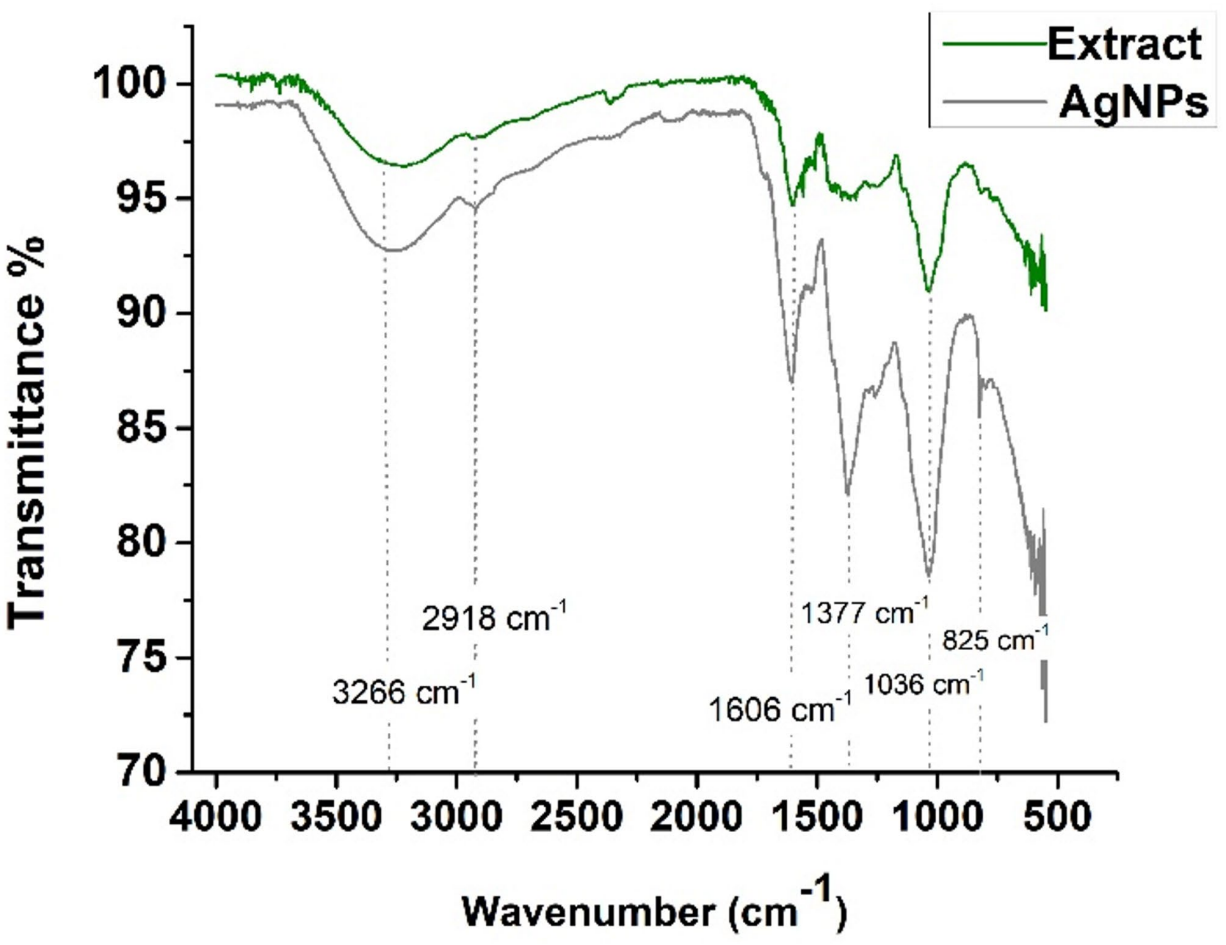
Comparative FTIR spectrum of AgNPs and *Vicia faba* seed coat extract indicating the presence of identical functional groups namely phenols and flavonoids.

The FTIR spectrum of the green synthesized silver nanoparticles using *V. faba* seed coat aqueous extract was compared with the FTIR spectrum of *V. faba* seed coat extract (Fig. 4).

The comparison revealed that there were noticeable peak shifts at wavenumber 3266 cm^− 1^ (O-H stretch) corresponding to phenols^35,36^, at 2918 cm^− 1^ (C-H stretch) corresponding to alkanes^36^, at 1606 cm^− 1^ (C = C) corresponding to aromatic ring stretching vibrations, linked to the deformation of aromatic rings commonly found in flavonoids and amino acids^37^. The 1377 cm^− 1^ peak may be attributed to CH bending owing to the presence of alkenes^38^. The peaks at 1036 and 825 cm^− 1^ were attributed to the C–N stretching vibrations of aromatic and aliphatic amines, indicative of the presence of complex phenolic compounds^39,40^. Furthermore, the disappearance of peaks was observed in the FTIR spectra of the *V. faba* seed coat extract at 1377 cm^− 1^.

### In vitro anticancer effect of AgNPs

**Fig. 5:**
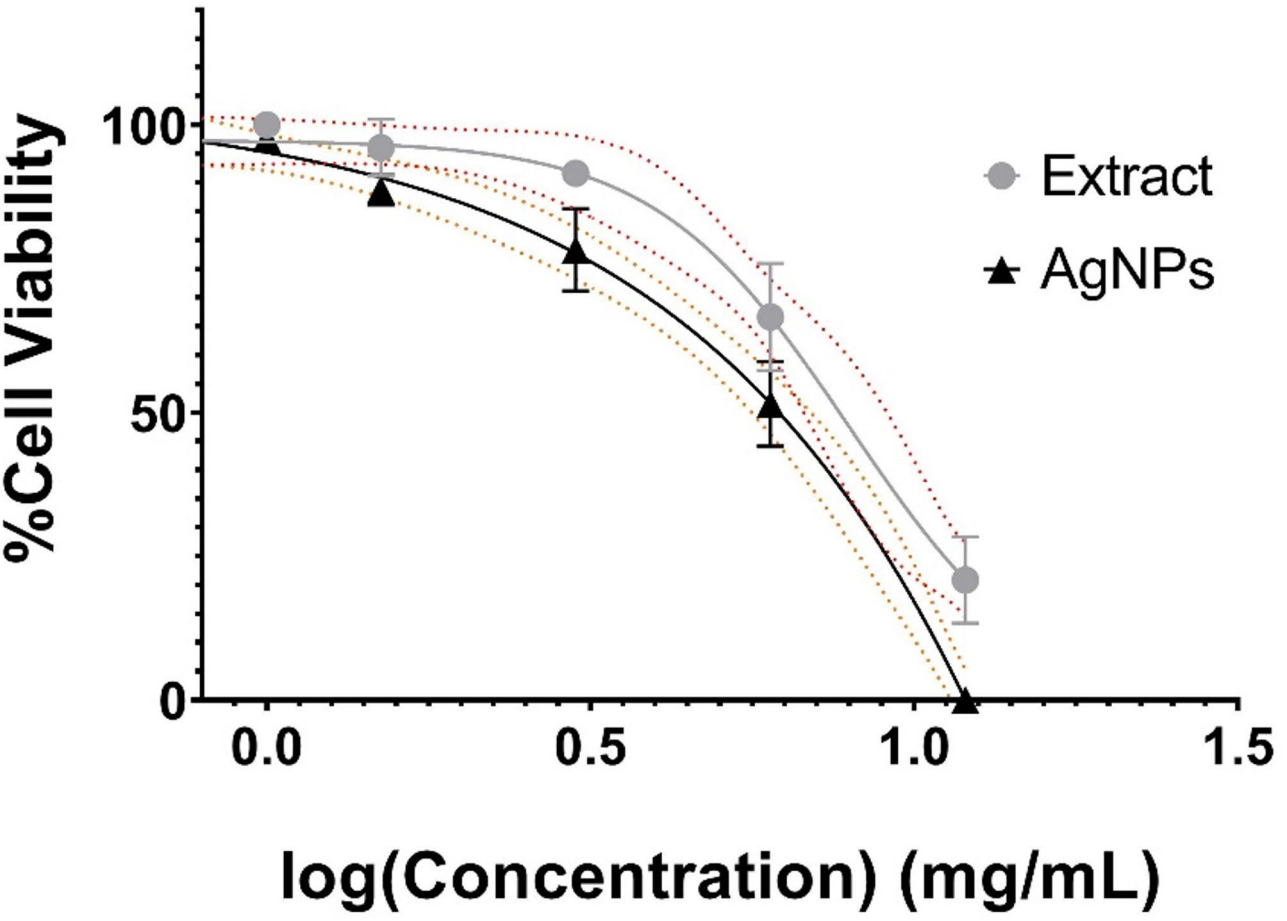
Cell viability of Jurkat cells treated with biosynthesized AgNPs and *Vicia faba* seed coat extract. Viability of Jurkat cells was determined 24 h after exposure to different concentrations of AgNPs and *Vicia faba* aqueous extract with a Trypan Blue exclusion assay.

A cell viability test was carried out to compare the effects of AgNPs and *V. faba* seed coat extract against Jurkat cells. The anticancer activity was found to be dose dependent, with the percentage of viability ranging from 96.59 to 51.49% at concentrations of 0.375 mg/mL to 3 mg/mL, respectively, whereas at 6 mg/mL there were no viable cells remaining. Additionally, aqueous extract decreased viability from 94.87% at 0.750 mg/mL to 29.03% to 12 mg/mL. After 24 h treatment the IC_50_ for extract and AgNPs were found to be 7.22 mg/mL and 2.27 mg/mL, respectively (Fig. 5). Similarly, the cytotoxic ability of *V. faba* beans extract against different cancer cells such as AGS (gastric), HT-29 (colon), BL13 (bladder), Hep G2 (liver) with IC_50_ values ranging from 1.04 to 2.03 mg/mL of crude extract^23^. In addition, AgNPs have been synthesized from other reducing agents using plants such as *Catharanthus roseus*^24^ and *Cucumis melo* L^32^ Leaf Extract have revealed some anticancer activity against Jurkat cell lines, but our treatment is more efficient^28,41^. Now, AgNPs synthesized from seed coat showed superior cytotoxicity than crude seed coat extract, this demonstrates the efficacy of the synergy between the properties of extract and nanoparticles.

**Fig. 6:**
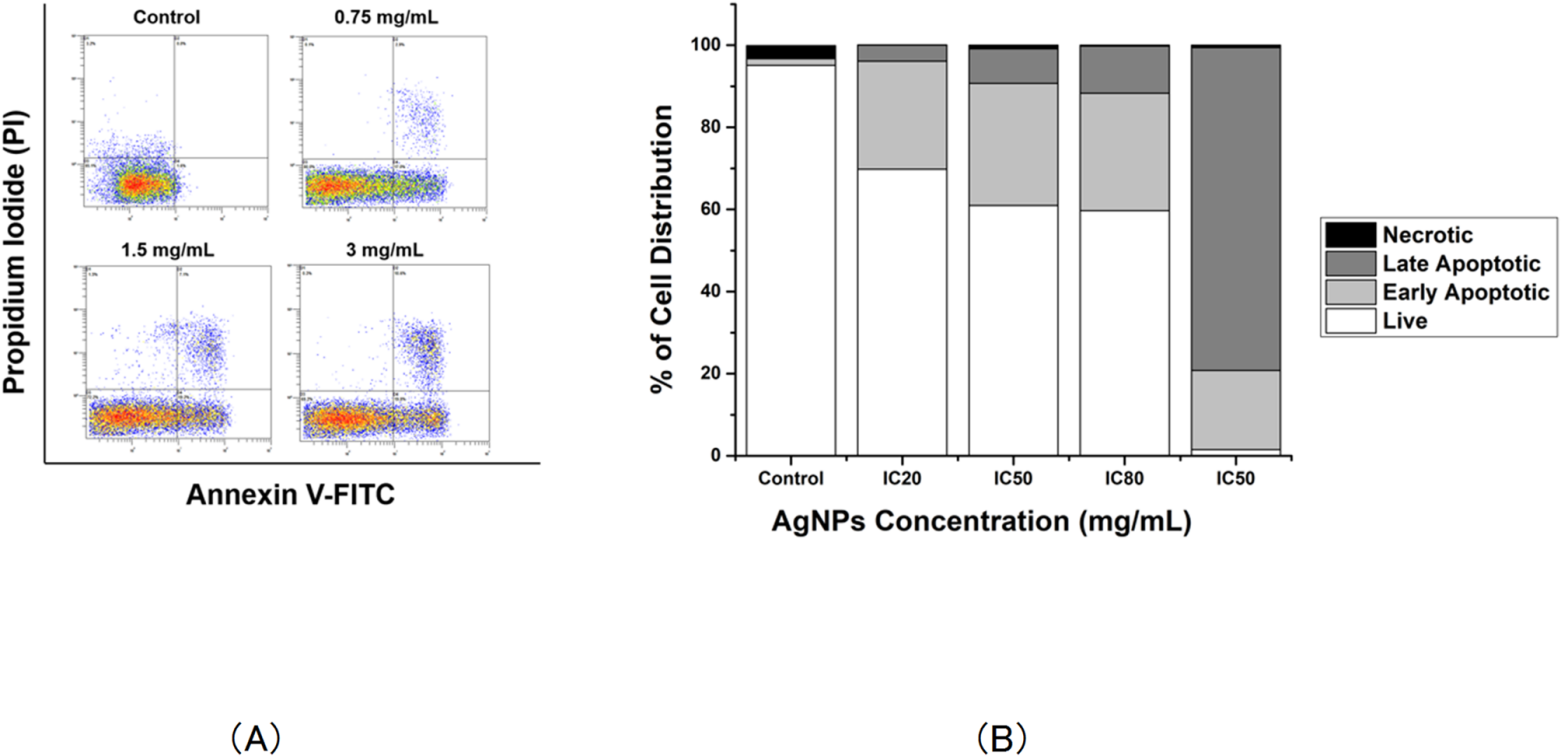
Apoptosis detection via Annexin-V/Propidium Iodide staining with flow cytometry. (**A**) Dispersion diagrams of cytometry data acquired for control, 0.75, 1.5, and 3 mg/mL AgNPs treatment. (**B**) Stacked column histogram of cell distribution in each apoptotic state for AgNPs.

Cytotoxic effect of AgNPs was also assessed through Annexin V/Propidium Iodide flow cytometry to evaluate AgNPs associated induction of apoptosis. The representative dot plots for the flow cytometric analysis comparing untreated cells and treated cells at 24 h showed an increase in the percentages of treated cells in early apoptosis and late apoptosis according to AgNPs concentration (Fig. 6A). According to the flow cytometry results, the control population revealed 95.1% viable cells, 1.6% entering apoptosis, and 3.2% necrotic cells. In contrast, cells exposed to AgNPs show significantly lower proportions of viable cells, corresponding to increased fractions of apoptotic and necrotic cells (Fig. 6B). This effect was strongly correlated with nanoparticles concentration, 3 mg/mL AgNPs decreased viable cells to 59.7% while the rate of necrotic cells was 11.5%. These results suggest that the antiproliferative effect of studied AgNPs on Jurkat cells results from induction of cell apoptosis.

### Cell cycle arrest

**Fig. 7:**
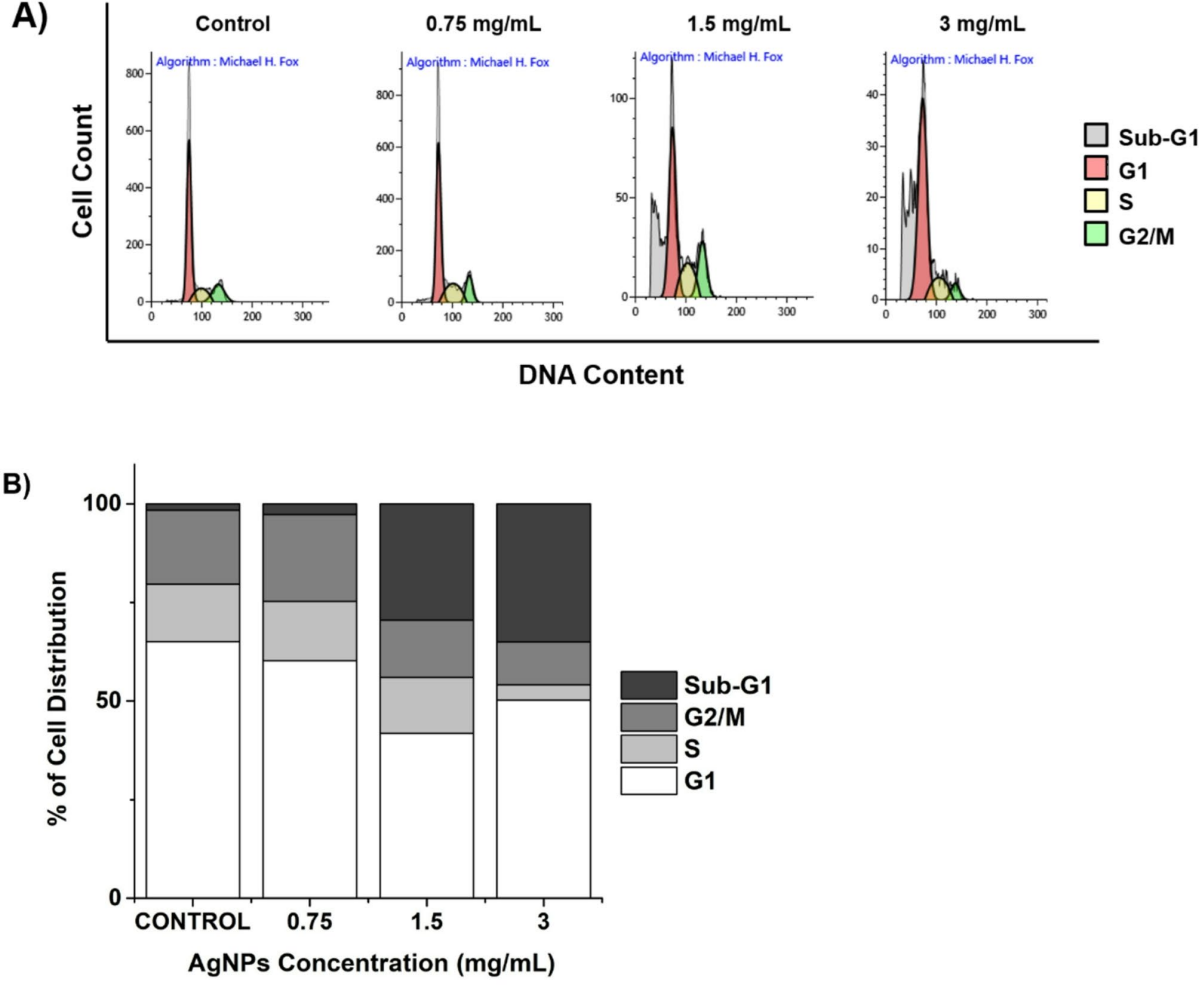
Effect of *V. faba* seed coat extract mediated silver nanoparticles in cell cycle progression of Jurkat cells. (**A**) Flow cytometry of cell cycle of untreated and treated cells. (**B**) Graphical representation of cell cycle proportions in each treatment.

The cell cycle arrest induced by AgNPs treatment in Jurkat cells is a critical observation, elucidating the impact of silver nanoparticles on cellular processes. Upon exposure to agents triggering apoptosis, a distinct subpopulation emerges preceding the G1 peak, denoted as the sub-G1 peak, attributed to the activation of endonucleases and subsequent DNA leakage from cells. Notably, necrotic cells exhibit no immediate reduction in DNA content, facilitating clear discrimination between necrotic and apoptotic cells. As delineated in Fig. 7A, the sub-G1 population, indicative of apoptotic cells, exhibits a concentration-dependent increase from 1.7% in the control group to 35% in cells treated with 3 mg/mL of AgNPs.

Regulation of the cancer cell cycle is a potential strategy in the development of anticancer drugs. Namvar et al. demonstrated a shift of cell distribution into the G2/M phase, which was enhanced by AgNPs^42^. As depicted in Fig. 7B, there is a noteworthy decrease in the percentage of cells in the G2/M phase after treatment with increasing concentrations of AgNPs. Our findings reveal an escalation in the sub-G1 population, corresponding to apoptotic cells, in accordance with the AgNPs concentration. Conversely, divergent studies have reported cell arrest in G1/S and G2/M phases^43,44^. These conflicting outcomes suggest inherent variances across distinct cell types. These conflicting results suggest inherent differences between cell types. Treatment with AgNPs induces cell cycle arrest through coordinated regulation of key proteins involved in cell cycle checkpoints and apoptosis. According to Meenakshisundaram et al. (2020)^45^, AgNPs synthesized with *Annona muricata* extract caused a significant decrease in the expression of cyclins D, E, and B, essential for G1/S and G2/M progression, respectively, along with activation of p53 and p21, central factors that arrest the cell cycle in response to genotoxic damage. At the same time, the inhibition of the anti-apoptotic protein Bcl-2 was observed, favoring intrinsic pathway-mediated apoptosis. Consistently, Singh et al. (2021) reported that AgNPs synthesized with *Carica papaya* extract promoted overexpression of p53 and p21, inhibited Bcl-2, and induced both G0/G1 arrest and activation of the apoptotic cascade. These results underline that the control of the cell cycle by AgNPs depends mainly on the activation of the p53/p21 axis and the modulation of the Bcl-2 balance, mechanisms that act in synergy to facilitate programmed death and disrupt cell proliferation.

## Conclusion

The biosynthesis of silver nanoparticles using *Vicia faba* seed coat extract (possessing different and improved properties from those of the seed) proved to be an environmentally friendly, efficient and effective method, generating mostly spherical nanoparticles, with low polydispersity and physicochemical properties such as stability and low aggregation, which are essential to control chemical reactions and make them suitable for biomedical applications. AgNPs synthesized from *V. faba* bean envelope extract showed strong cytotoxic activity against Jurkat leukemic cells, inducing apoptosis and cell cycle arrest in sub-G1 phase in a concentration-dependent manner. These effects are attributed to the synergy between the bioactive compounds of the plant extract and the intrinsic properties of the nanoparticles, which positions this system as a promising strategy for the development of alternative and sustainable anticancer agents.

## Data Availability

All data generated or analyzed during this study are included in this published article.
